# Emerging Research on Endocrine Disruptors

**Published:** 2007-01

**Authors:** David A. Schwartz, Kenneth S. Korach

**Affiliations:** Director, NIEHS and NTP, E-mail: david.schwartz@niehs.nih.gov; Director, Environmental Disease and Medicine Program, E-mail: korach@niehs.nih.gov

For more than three decades, the NIEHS has been one of the recognized leaders in the world in the study of endocrine disruptors, substances that mimic or alter hormonal effects in the body. In the 1970s, NIEHS scientists pioneered research on reproductive toxicity and discovered the hormonal toxicity of diethylstilbestrol (DES), a drug that for years had been prescribed to women to prevent miscarriage, but that was shown to later cause cancer and infertility in some of the children of these mothers. Clinical researchers used observations from this basic science to replicate in animal models what was being seen in patients, ultimately leading to the discontinued use of DES in pregnant women. Now NIEHS investigators are demonstrating that exposure to endocrine disruptors such as DES during critical developmental stages can actually reprogram the genomes of offspring to cause inappropriate gene expression, resulting in increased susceptibility later in life to diseases such as cancer, as well as to reproductive, metabolic, and developmental disorders. The ability of an environmental compound to induce an epigenetic disease state spanning several generations has significant implications for the study of disease origins and consequences of exposure. For this reason the study of endocrine disruptors continues to be a research priority at the NIEHS.

The NIEHS has been at the forefront of basic research to understand the toxicology of reproductive and developmental health. This is particularly important in the case of endocrine disruptors, which act by multiple mechanisms. To discern whether the toxicity of such chemicals is due to hormonal activity or some other activity of the compounds, researchers must first understand how estrogens and the estrogen receptor work at a basic level. Seminal work by NIEHS researchers has identified new sites of action and biological activities, developed animal models to show how estrogens work, and shown how environmental factors might disrupt them. Recent studies have found a contributing role for estrogen receptor signaling in the ovary with regards to ovulation, providing the potential for identifying its effect on subfertility or infertility.

Researchers in the NTP Center for the Evaluation of Risks to Human Reproduction (CERHR) have evaluated the endocrine-disrupting effects of seven phthalates, chemicals used in plastics manufacture, and the phytoestrogen genistein, found in soy. The phthalate evaluations have had broad impact, guiding regulatory agencies in making decisions on these chemicals. For example, the center’s report was in part the basis of a Consumer Product Safety Commission recommendation that certain phthalates be removed from mouthing toys (such as teethers and rattles) due to concerns about children ingesting these chemicals. In response to a CERHR report on another phthalate used in plastic tubing, the Food and Drug Administration issued guidance pointing out the potential danger to newborns and infants receiving treatments with medical devices containing di(2-ethylhexyl) phthalate. The evaluation of genistein, which included many NIEHS studies on early developmental exposures, concluded that it produces reproductive and developmental toxicity, which has prompted recognition and further research on the potential health effects of environmental estrogens. In March 2007, the CERHR will convene an expert panel to determine whether exposure to bisphenol A, found in many plastics and in the linings of steel food cans, is a hazard to human development or reproduction.

The NIEHS will move research on endocrine disruptors forward by developing sensitive markers of exposure, early biological response, and genetic susceptibility. New research is considering the role of endocrine-disrupting chemicals in conditions such as obesity. NIEHS epidemiologists are also looking at global populations to determine whether environmental estrogens affect fertility, reproduction, or the onset of puberty. For example, researchers have found that chemicals such as polychlorinated biphenyls and DDT may affect body size and advance some of the early changes in girls at puberty. NIEHS scientists are developing an animal model to evaluate the toxic effects of environmental agents on the prostate. And other ongoing investigations are elucidating the endocrine-disrupting effects of naturally occurring substances found in common health and beauty products. The NIEHS is continuing to collaborate with Duke University Medical Center and the University of North Carolina Medical School to support joint reproductive studies, some of which focus on the effects of endocrine disruptors, as well as working to build new research partnerships in this area. Studies like these are at the heart of the new NIEHS Strategic Plan because they compel us to consider how the research we conduct fits into the complex puzzle of improving human health.

Although we have made significant progress in the study of endocrine disuptors, we’re still a long way from understanding all of the health effects of these ubiquitous chemicals. Until we do, the NIEHS will remain committed to this important area of research, with its ramifications for generations to come.

## Figures and Tables

**Figure:**
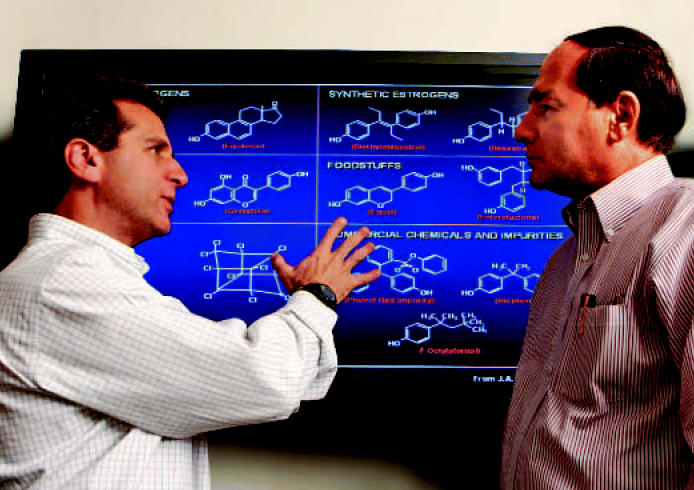
(left to right) David Schwartz and Ken Korach

